# Prostaglandin-Endoperoxide Synthase 2 (PTGS2) Expression Is Increased in Chronic Asymptomatic Orchitis in the Dog

**DOI:** 10.3390/vetsci13101110

**Published:** 2026-10-17

**Authors:** Eva-Maria Packeiser, Hanna Körber, Judith Morawietz, Sandra Goericke-Pesch

**Affiliations:** Unit for Reproductive Medicine, Clinic for Small Animals, University of Veterinary Medicine Hannover, Foundation, 30559 Hannover, Germany

**Keywords:** infertility, dog, inflammation, chronic asymptomatic orchitis, PTGS2, StAR

## Abstract

Chronic asymptomatic orchitis (CAO) in dogs lacks effective treatment options. To elucidate the involvement of prostaglandin endoperoxide synthase 2 (PTGS2), associated with inflammation and physiological steroidogenesis, testicular PTGS2 and StAR gene (via RT-qPCR) and protein expression (via immunohistochemistry) were analyzed in 11 canine CAO samples and compared to five normospermic control group (CG) samples. The percentage of immunopositive area (PIA) and mean gray scale (MGS) intensity were evaluated in the interstitial and tubular compartments. In CAO, *PTGS2* and *StAR* gene expression levels were significantly increased compared to CG (*p* < 0.001 and *p* = 0.0034). Leydig cells and some peritubular cells stained immunopositive for PTGS2 and StAR and Sertoli cells positive for PTGS2. PIA and MGS for PTGS2 differed significantly between groups in the interstitial (PIA: *p* < 0.0001; MGS: *p* = 0.0051) and tubular (PIA: *p* = 0.0006; MGS: *p* = 0.0413) compartments. For StAR, PIA, but not MGS, was significantly higher in CAO (*p* = 0.0010). Except for *PTGS2* gene expression, higher degrees of spermatogenetic impairment correlated with more prominent expression changes. This study demonstrates an association between increased PTGS2 expression and CAO. Further research is needed to investigate potential clinical benefits of selective PTGS2 inhibitors.

## Simple Summary

Chronic asymptomatic orchitis (CAO) is the most common reason for infertility in male dogs and currently lacks effective treatment options. Prostaglandin endoperoxide synthase 2 (PTGS2) plays an important role in inflammation and, together with StAR, in the synthesis of testosterone. To elucidate the involvement of PTGS2 in the development, maintenance, and progression of CAO, testicular PTGS2 and StAR expression were examined at the gene and protein levels in 11 canine CAO samples and compared to five normospermic controls (CG). The percentage of the immunopositive area (PIA) for PTGS2 and StAR and the mean gray scale (MGS) were evaluated in the sperm- and hormone-producing compartments of the testes. In CAO, expression at both the gene and protein levels was significantly increased. Only the MGS of StAR was not significantly altered in the hormone-producing compartment. Furthermore, for all parameters except *PTGS2* gene expression, higher degrees of spermatogenic impairment were accompanied by more prominent expression changes. This study demonstrates an association between increased PTGS2 expression and CAO. Further research is needed to investigate the potential clinical benefits of selective PTGS2 inhibitors.

## 1. Introduction

Infertility due to azoospermia, the complete absence of spermatozoa in the ejaculate, represents, at 34.8%, the most frequent clinical finding in infertile male dogs [1]. As in humans [2], canine azoospermia is mainly related to a disruption of spermatogenesis [3]. Immune cell infiltrations, mainly of lymphocytes, are regularly detected in testicular biopsies from infertile human [4,5,6] and canine patients [7,8], recently resulting in the common diagnosis of chronic asymptomatic orchitis [6,7].

Although the cause for induction of chronic asymptomatic orchitis (CAO) still remains unknown, in the canine testis, an early and a late form with varying degrees of altered spermatogenesis have already been identified [7]. Plasma cells and T cells invade the testicular parenchyma and cross the disrupted blood testicular barrier [9], leading to impaired spermatogenesis and sustained inflammation [7,8,10]. This ongoing inflammation is likely the reason why currently CAO results in permanent infertility without any available treatment options, although spermatogonial stem cells remain present—at least in some tubules [11].

Prostaglandins play an important role in inflammation and pain [12,13], and the prostaglandin endoperoxide synthase (PTGS) 2, the key enzyme [14,15,16], was shown to be expressed in rat testis with experimentally induced autoimmune orchitis (EAO) [17] and infertile human patients with disturbed spermatogenesis [18,19]. The detection of PTGS2 in the healthy testicular tissue of various other species, such as Syrian hamster [20], mouse [21], rat [22,23], as well as dog [24,25], indicates that prostaglandins are, however, not only associated with inflammation but also involved in testicular development, steroidogenesis, and spermatogenesis in various species [20,21,22,23]. Namely, PTGS2 and prostaglandins have been shown to regulate the transcription of the *steroidogenic acute regulatory protein* (*StAR*), the bottleneck of steroidogenesis [26,27], in murine Leydig cell cultures [28]. This hypothesis is strengthened by the co-expression of PTGS2 and StAR in murine and canine Leydig cells [25,28,29,30]. Beyond Leydig cells, Sertoli cells and some peritubular cells express PTGS2 in the Syrian hamster [20], mouse [21], rat [22], dog [24,25], and human [18] testis. In addition, mast cells expressed PTGS2 in testicular biopsies of infertile human patients with mixed atrophy [31]. Further, the number of mast cells was significantly increased in infertile men with disturbed spermatogenesis, germ cell arrest syndrome, Sertoli-cell-only-syndrome, and mixed atrophy [32] compared to patients with normal spermatogenesis [31,33,34,35]. Mast cells likely contribute to disruption of spermatogenesis [35] not only through PTGS2-driven inflammation but also via tryptase [36], a serine protease that induces fibrosis [32,37], which plays an important role in spermatogenesis disorders [38,39]. In healthy canine testis, mast cells are rarely detected [40]; however, as canine CAO is associated with increased fibrosis [7], we postulate that tryptase-expressing mast cells are involved in the pathogenesis of the disease. In summary, PTGS2 as the key enzyme of inflammation is suspected to be involved in CAO pathology. Its general influence on steroidogenesis has been demonstrated [28] in vitro in mouse Leydig cells but has not yet been investigated in the context of CAO. Furthermore, PTGS2 is also expressed by mast cells, which is why we aimed to identify and characterize these cells.

Against this background, the purpose of this study was to investigate PTGS2 expression in CAO-affected canine testicular biopsies with varying degrees of spermatogenic impairment to elucidate the role of PTGS2 in CAO pathophysiology. To determine whether increased PTGS2 expression compared to healthy tissues is associated with the presence of mast cells in CAO samples, tryptase staining was conducted. To further specify the overall influence of PTGS2 on steroidogenesis, StAR expression was also evaluated.

## 2. Materials and Methods

### 2.1. Study Design and Sample Collection

Sixteen sexually mature clinically healthy male dogs were included in this study: Eleven dogs suffered from non-obstructive azoospermia (confirmed by repeated semen samples) and were presented with a history of infertility at the clinic for further diagnostics. Briefly, clinical and andrological examinations including semen analysis and ultrasound of the genital tract, determination of alkaline phosphatase in seminal plasma, bacteriological examination of the ejaculate, serological testing for *Brucella canis*, and endocrine analysis confirmed the diagnosis of non-obstructive azoospermia with no indication of a pretesticular or infectious origin [7]. Testicular biopsies of both testes were obtained for diagnostic purposes under general anesthesia as scissors biopsies. Histological assessment revealed CAO in all respective dogs (*n* = 11) [7,8]. For further differentiation, the spermatogenic maturation arrest was characterized as previously described [7], namely as “early arrest” (*n* = 5), in which spermatogenesis is arrested mainly at the spermatogonial level, and as “late arrest” (*n* = 6), with further developed germ cell stages present. Dogs with CAO had a mean age of 6.5 ± 1.7 years (range: 4.5–9.5 years, Supplementary Table S1), a mean body weight of 15.7 ± 9.6 kg (range: 5.0–18.0 kg) and belonged to nine different breeds (Collie: *n* = 3; Cairn Terrier, Miniature Poodle, Labrador Retriever, Icelandic Sheepdog, Welsh Corgi Pembroke, Coton de Tulear, Beagle, and Jack Russel Terrier: *n* = 1 each).

The remaining five dogs were considered the healthy control group (CG). Normospermic semen samples with all parameters (motility, viability, morphology, and total sperm count) within the reference range [41] had been collected from these dogs. Dogs in the CG (*n* = 5) were a mean of 4.5 ± 4.0 years old (range: 0.9–9.9 years, Supplementary Table S1) and had a mean body weight of 7.6 ± 3.1 kg (range: 3.6–11.5 kg). The animals belonged to four different breeds (*n* = 1 each: Havanese, Maltese, Boston Terrier, Chihuahua); one dog was mixed breed. Testicular tissue samples from the CG had been collected after surgical castration, which was performed at the owner’s request due to non-medical reasons.

All owners agreed to the use of testicular tissue samples for research purposes. As only leftover tissue was used, no ethics approval was needed. Tissue samples of the right testis were immersed in RNAlater^®^ (Ambion Biotechnologie, Wiesbaden, Germany) and stored at −80 °C for mRNA extraction. Samples of both testes (CAO) or of the right testis (CG) were fixed in Bouin’s solution and embedded in paraffin for histology and immunohistochemistry as previously described [42]. The same dogs were used for gene and protein expression analyses.

### 2.2. RNA Extraction, Reverse Transcription and Quantitative Reverse Transcriptase Polymerase Chain Reaction (RT-qPCR)

Total RNA was isolated from the right testicular tissue samples of dogs with CAO (*n* = 11) and CG dogs (*n* = 5) using a TRIzol-based protocol (Thermo Scientific, Waltham, MA, USA). One dog from the CAO group was excluded from the mRNA analysis due to repeatedly low RNA yields during extraction. Full-length first-strand cDNA synthesis was performed by using 200 ng/µL RNA and the RevertAid First Strand cDNA Synthesis Kit (#K1622, Thermo Scientific), according to the manufacturer’s protocol including DNase treatment and nuclease-free water as a non-template control. Previously established primer sets for *PTGS2* and *StAR*, as well as for the two reference genes *GAPDH* (*glyceraldehyde-3-phosphate dehydrogenase*) and *ACTB* (*β-actin*), were purchased from Microsynth (Balgach, Switzerland; Table 1). Specific binding of the primers in the canine testis was previously ensured by sequencing the amplicons [25,29]. For RT-qPCR, 3 µL of cDNA (dilution 1:10) was added to 8 µL of FastStart Essential DNA Green Master (Roche Diagnostics, Mannheim, Germany), 1 µL each of the forward and reverse primers (10 pmol), and 2 µL of nuclease-free water. The RT-qPCR conditions were 95 °C for 10 min, followed by 35 cycles of 95 °C for 10 s, 60 °C for 10 s, 72 °C for 10 s, and a melting curve. Non-template controls were included in every assay. All samples were run in triplicate using a LightCycler^®^96 real-time PCR system (Software release 1.5.0, Version 1.5.0.39, Roche Diagnostics). Primer efficiencies were calculated using a relative standard curve derived from a triplicate RT-qPCR run of a two-fold dilution series (1:2–1:128) of pooled cDNA samples. Pooled CG samples were included in each batch as a calibrator. For calculation of the relative gene expression ratio, an efficiency-corrected relative quantification according to Pfaffl [43] was performed. As *GAPDH* showed the slightest variation in mRNA expression compared to *ACTB*, *GAPDH* was chosen as the reference gene for calculations.

### 2.3. Immunohistochemistry and Evaluation of PTGS2, StAR and Tryptase Staining

Immunohistochemistry for PTGS2 was performed as previously described, and specific antibody binding in the canine testis was verified by Western blotting [25]. Serial sections (2 µm) from paraffin-embedded tissues (CAO: *n* = 11 dogs, left and right samples; CG: *n* = 5, only right sample) were deparaffinized, boiled in citrate buffer (pH = 6), incubated with 3% hydrogen peroxide in methanol, and incubated with 10% horse serum to block nonspecific binding (S-2000, Vector Laboratories, Newark, CA, USA). Blocking was performed in ICC buffer (1.2 g Na_2_HPO_4_, 0.2 g KH_2_PO_4_, 0.2 g KCl, 8.0 g NaCl, 3 mL Triton ad 1 L), after which, sections were covered with the PTGS2 monoclonal antibody (Clone CX229, Cayman Chemicals, Ann Arbor, MI, USA, dilution 1:100 corresponding to 0.005 mg/mL) overnight at 4 °C. On the following day, samples were incubated with the secondary antibody (BA-2000, Vector Laboratories, dilution 1:200). Immunopositive signals were visualized by an immunoperoxidase method according to the manufacturer’s instructions (VECTASTAIN PK-6100 Rabbit IgG Elite ABC Kit and Vector Nova-RED Substrate Kit SK-4800, Vector Laboratories). Slides were counterstained with hematoxylin. An irrelevant mouse IgG antibody (I-2000, Mouse IgG, Vector Laboratories) at the respective protein concentration served as the isotype control.

StAR immunostaining was performed with slight modifications similar to earlier descriptions, in which specific antibody binding was also confirmed by Western blotting [29]. Phosphate-buffered saline (PBS) was used for washing, while 3% bovine serum albumin (VWR Life Science, Solon, OH, USA) and 10% goat serum (S-1000, Vector Laboratories) in PBS buffer were used for blocking. The slides were incubated with a polyclonal antibody against a murine StAR peptide (dilution 1:2000; kindly donated by Prof. D.M. Stocco [29,44]) overnight at 4 °C. On the following day, slides were washed twice with PBST (PBS with 0.1% Tween 20) and once with PBS followed by incubation with SuperVision-2 HRP Enhancer (DCS, Innovative Diagnostik Systeme, Hamburg, Germany). Immunopositive signals were visualized with DAB (DCS). Here, an irrelevant rabbit IgG antibody (I-1000, Rabbit IgG, Vector Laboratories) served as the isotype control.

Tryptase immunostaining was conducted as described for PTGS2, but using a tryptase monoclonal antibody (Clone AA1, Dako Denmark, Glostrup, Denmark, dilution 1:100). Specific antibody binding in canine mast cells had previously been confirmed by flow cytometry, immunofluorescence, and immunocytochemistry [45,46]. On the following day, preparation was performed as described for StAR (SuperVision-2 HRP Enhancer, HRP Polymer and DAB), according to the manufacturer’s instructions. A canine mast cell tumor served as the positive control. Evaluation of immunopositive signals for tryptase in serial sections was descriptive and comparative to PTGS2 staining using the Olympus BX41TF microscope (Shinjuku, Japan) at 200× and 400× magnification. All stainings for PTGS2, StAR, and tryptase, were performed in single batches to avoid bias. Negative controls using buffer only were included.

For computer-assisted image analysis of the slides, the ImageJ Fiji software [47] (https://fiji.sc, accessed on 10 July 2026, version 2.18.0) was used. To separate and differentiate specific expression changes within individual compartments, immunopositive signals were evaluated specifically for the interstitium (PTGS2 and StAR) and the tubules (PTGS2). Since StAR was only expressed in the interstitial compartment, the immunopositive area (PIA) and the staining intensity (mean gray scale, MGS) of the interstitial tissue (PTGS2 and StAR) and tubules (PTGS2) were quantified [40]. For the interstitial compartment, 20 randomly chosen images (10 for each side in CAO) were taken at 400× magnification (Olympus BX41TF) using the Olympus cellSens Dimension software (version 2.1). Similarly, for the tubular compartment, images from 30 randomly selected tubules (15 right/left each in CAO) were taken at 200× magnification. Depending on the area of interest, either the interstitial tissue including peritubular cells and Leydig cells, or the seminiferous tubules (area within the basement membrane) were set as the region of interest, and blood vessels and artifacts were cropped. All images were converted to grayscale pictures, with pixel gray values ranging from zero (white) to 255 (black). Individual thresholds defining immunopositive staining were set for the interstitial tissue and tubules, separately for PTGS2 and StAR. To evaluate the MGS, all positive pixels above the threshold were analyzed. The PIA was determined by binarized grayscale images applying the individual thresholds [40].

### 2.4. Statistical Analysis

GraphPad Prism 7 software (GraphPad Software, La Jolla, CA, USA) and Microsoft Excel (Version 16.109.2, Microsoft, Redmond, WA, USA) were used for statistical analysis. Values at a level of *p* < 0.05 were considered statistically significant.

The primary objective was to identify significant differences between the CAO and CG groups regarding *PTGS2* and *StAR* mRNA and protein expression. In a subsequent analysis, the CAO group was subdivided into early and late spermatogenic arrest, and groupwise comparisons were conducted. A power analysis based on gene expression data of *StAR* in hormonally downregulated and control testes [29] for comparing three groups in a one-way analysis of variance (ANOVA) with a significance level (alpha) = 0.05 (two-tailed), a target power = 0.8, a standardized effect size = 1.207, and a common standard deviation for each group = 0.4 resulted in a required sample size of *n* = 4 per group with an achieved power of 0.889.

The Shapiro–Wilk test detected no deviation from a normal distribution (*p* < 0.3) for the mRNA expression ratios of *PTGS2* and log-transformed *StAR* data for all groups. Consequently, CAO group data were compared with the CG using an unpaired *t*-test with Welch’s correction (utilizing log10-transformed data in the case of *StAR*). After subdividing the CAO group into early and late arrest, groups were compared using a one-way ANOVA, (with log10-transformed data for *StAR*), followed by Tukey’s multiple comparisons test adjusted for multiple testing.

PTGS2 (tubular and interstitial) and StAR (interstitial) protein expression levels were examined concerning PIA and MGS via objective computer-assisted analysis. To rule out side-specific effects within the CAO group, left and right testes were initially compared. No deviations from normal distribution (*p* < 0.2) were indicated by the Shapiro–Wilk test for the left and right testes with the CAO group across all PTGS2 data sets (PIA and MGS, tubular and interstitial compartments), as well as for StAR PIA in the interstitium. Subsequent paired *t*-tests revealed no significant differences between both testes. Likewise, the Wilcoxon signed-rank test did not detect a significant difference in interstitial StAR MGS between the left and right testes (not normally distributed according to the Shapiro–Wilk test). Therefore, the PIA and MGS values of the left and right testes of the individual CAO dogs were averaged.

Initially, all dogs with CAO were compared to the CG. These pairwise comparisons were conducted using unpaired *t*-tests (after log10 transformation of both interstitial PIA and MGS for PTGS2) after the Shapiro–Wilk test had indicated no deviation from normal distribution (*p* < 0.2). Subsequently, the CAO group was further differentiated into early and late arrest of spermatogenesis. According to the Shapiro–Wilk test, all groups were normally distributed (after log10 transformation of interstitial PIA for both PTGS2 and StAR). Therefore, groupwise comparisons were conducted using a one-way ANOVA with a subsequent Tukey’s post hoc test adjusted for multiple testing.

## 3. Results

### 3.1. PTGS2 mRNA and Protein Expression

**Figure 1:**
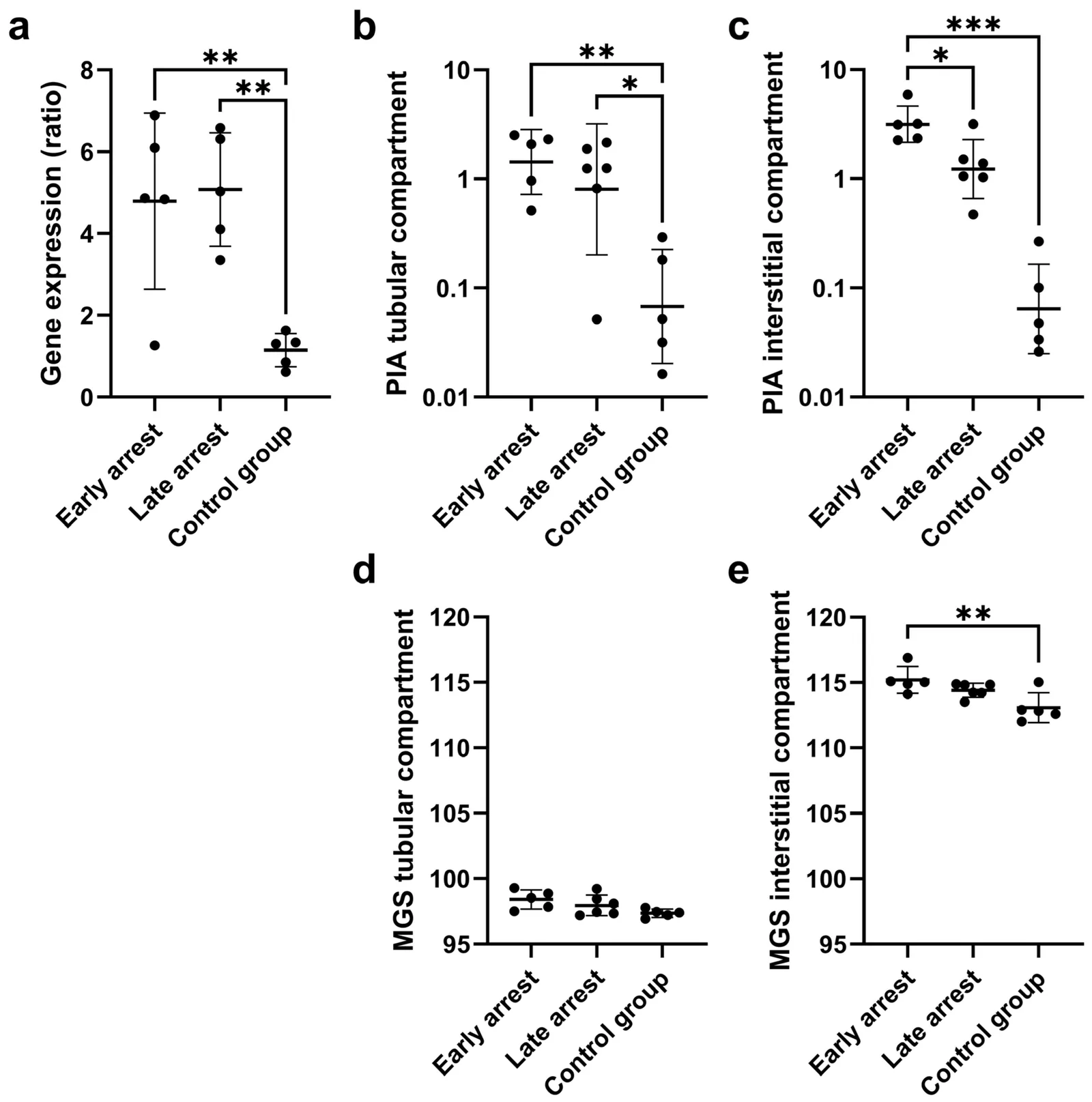
Testicular PTGS2 expression in dogs with chronic asymptomatic orchitis (CAO), divided into early-arrest and late-arrest spermatogenesis groups and in normospermic dogs in the control group (CG). (**a**) mRNA expression ratios, (**b**–**e**) protein expression within the tubular (**b**,**d**) and interstitial compartment (**c**,**e**). Results are presented as arithmetic mean with standard deviation for PTGS2 gene expression (**a**) and mean gray scale (MGS) (**d**,**e**) and as geometric mean with geometric standard deviation for percentage of immunopositive area (PIA) (**b**,**c**). Asterisks indicate significant differences according to Tukey’s test: * *p* < 0.05; ** *p* < 0.01, *** *p* < 0.001.

The *PTGS2* mRNA expression ratio was significantly higher in the CAO group than in the CG, as determined by the unpaired *t*-test (Table 2). After subdivision into the early- and late-arrest groups, a one-way ANOVA identified significant group differences (*p* = 0.0022), with higher gene expression in both the early-arrest (*p* = 0.0061) and late-arrest groups (*p* = 0.0036) compared with the CG (Figure 1).

**Figure 2:**
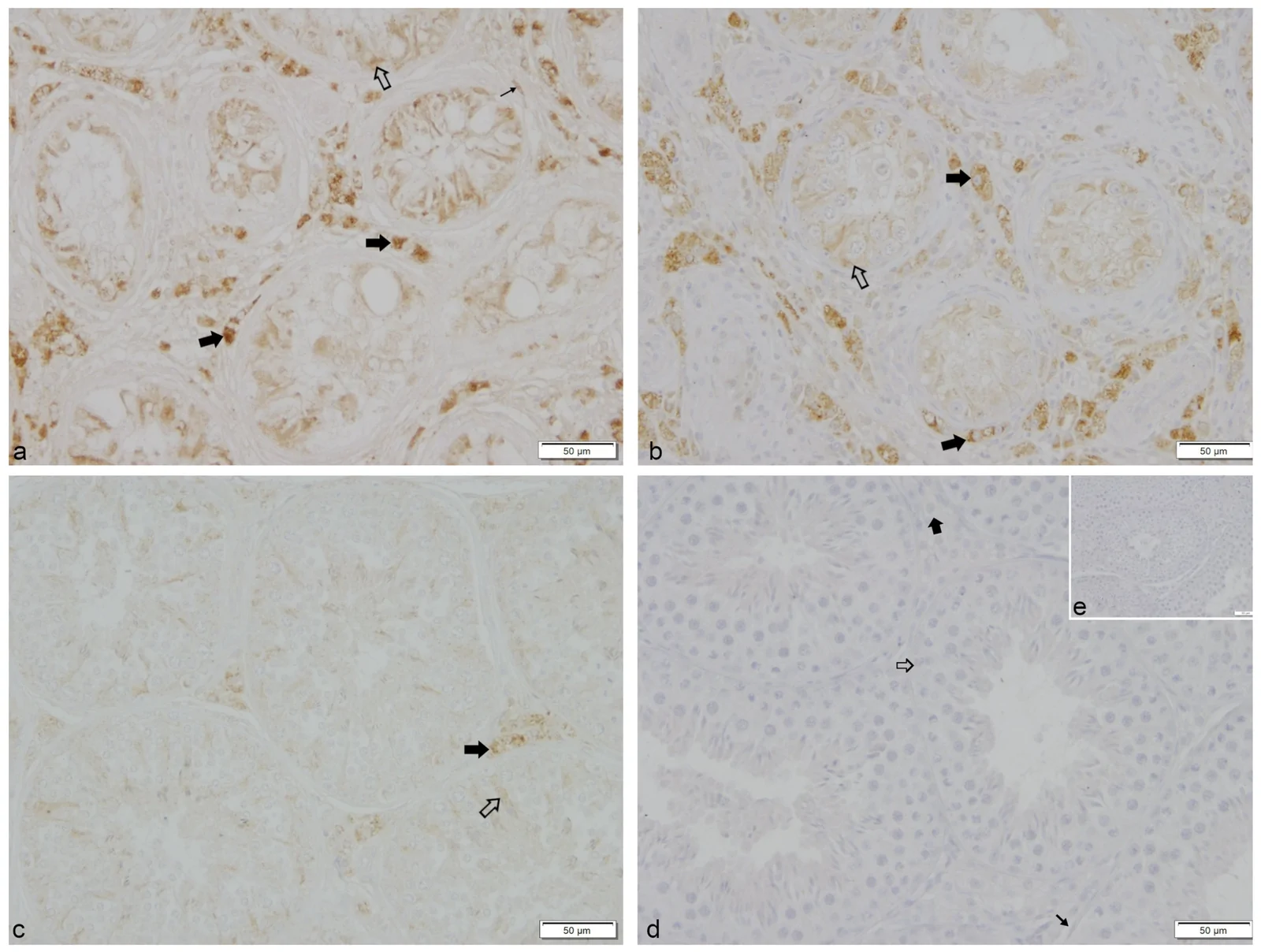
Immunostaining for PTGS2 in testicular samples from dogs with chronic asymptomatic orchitis (CAO) (**a**,**b**) and from normospermic dogs in the control group (CG) (**c**). (**a**) CAO—early spermatogenic arrest, (**b**) CAO—late arrest, (**c**) CG, (**d**) isotype control, (**e**) negative control (shown as an inset); solid arrows (➡) indicate Leydig cells, open arrows (⇨) indicate Sertoli cells, and long arrows (→) indicate peritubular cells ((**a**–**e**) 200× magnification).

Specific immunopositive staining for PTGS2 was observed in the cytoplasm of Sertoli cells and Leydig cells in all samples (Figure 2). The CG (Figure 2c) showed a uniform distribution of the immunopositive signal in the cytoplasm of Sertoli cells extending from the basement membrane to the lumen. In the CAO group (Figure 2a,b), the PTGS2 staining in Sertoli cells was irregular and diffuse within the whole sample. Additionally, some peritubular cells stained immunopositive in both groups (Figure 2).

Similar to the mRNA levels, PTGS2 PIA and MGS within the tubules and in the interstitial compartment were larger in the CAO group samples compared to CG (Table 2). Likewise, the subgroups (early arrest, late arrest, CG), differed significantly regarding PTGS2 PIA in both the tubular (*p* = 0.0084) and the interstitial compartment (*p* = 0.0007, both by ANOVA), with early and late spermatogenic arrest showing a higher PIA compared with CG (Figure 1b,c). When comparing the subgroups, however, MGS was significantly higher only in the early-arrest group compared to the CG in the interstitial compartment (ANOVA, *p* = 0.0091; Figure 1e).

### 3.2. StAR mRNA and Protein Expression

**Figure 3:**
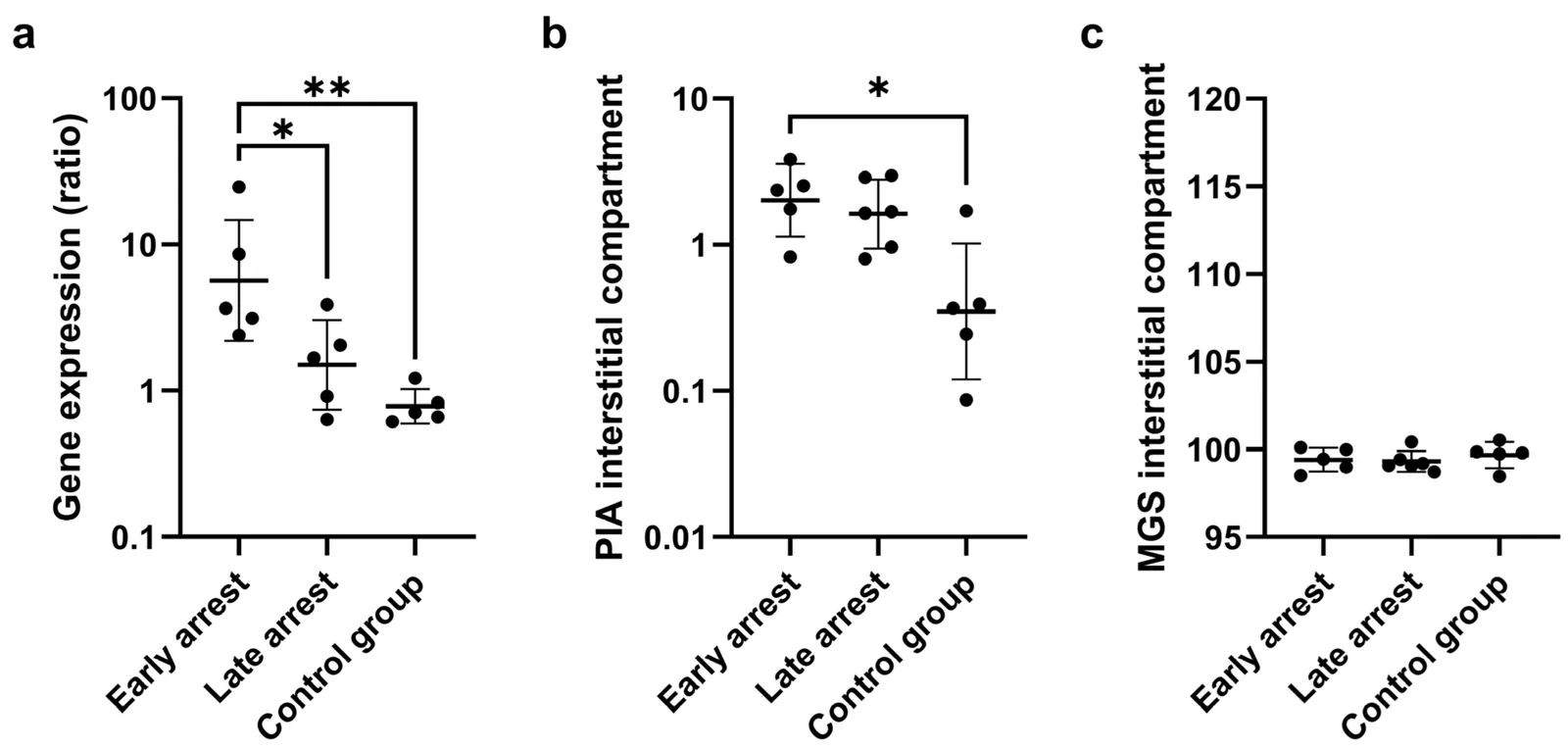
Testicular StAR expression in dogs with chronic asymptomatic orchitis (CAO), divided into early- and late-arrest spermatogenesis groups, and in normospermic dogs in the control group (CG). (**a**) mRNA expression ratios, (**b**,**c**) protein expression within the interstitial compartment. Results are presented as geometric mean with geometric standard deviation for gene expression (**a**) and percentage of immunopositive area (PIA) (**b**) and as arithmetic mean with standard deviation for mean gray scale (MGS) (**c**). Asterisks indicate significant differences according to Tukey’s test: * *p* < 0.05; ** *p* < 0.01.

The StAR mRNA expression ratio was significantly higher in the CAO group compared with the CG (Table 3). This was also apparent when comparing both stages of spermatogenic arrest (ANOVA, *p* = 0.0025; Figure 3a), although the ratio did not differ significantly between the late-arrest group and the CG (Figure 3a).

**Figure 4:**
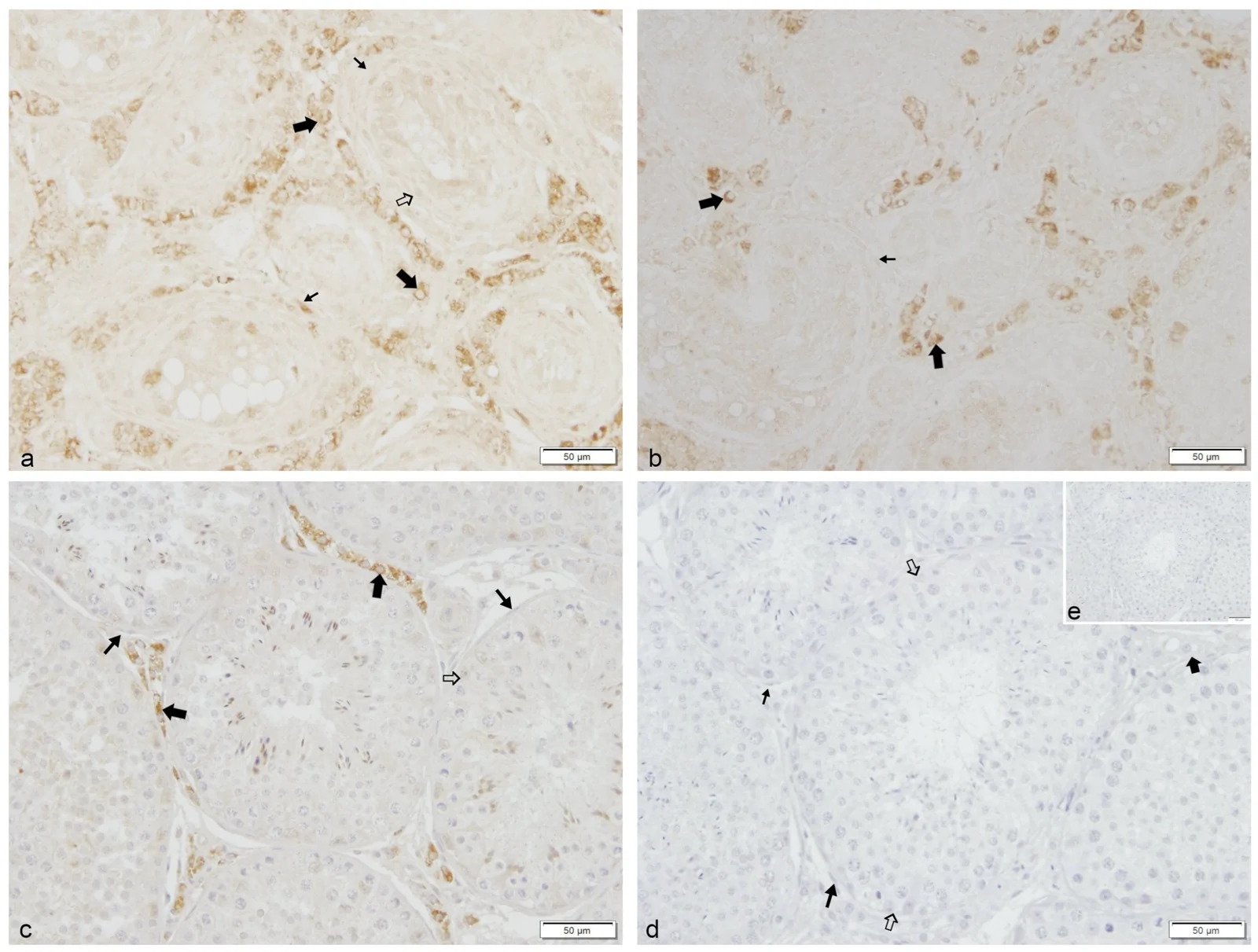
Immunostaining for StAR in testicular samples from dogs with chronic asymptomatic orchitis (CAO) (**a**,**b**) and from normospermic dogs in the control group (CG) (**c**). (**a**) CAO—early spermatogenic arrest, (**b**) CAO—late arrest, (**c**) CG, (**d**) isotype control, (**e**) negative control (shown as an inset); solid arrows (➡) indicate Leydig cells, open arrows (⇨) indicate Sertoli cells, and long arrows (→) indicate peritubular cells ((**a**–**e**) 200× magnification).

Specific immunopositive staining for StAR was observed in the cytoplasm of Leydig cells and some peritubular cells in both the CAO and CG samples (Figure 4). Computer-assisted objective evaluation revealed a significantly higher PIA in the interstitial compartment in the CAO group compared to the CG (*p* = 0.0010, Table 3). In contrast, the MGS did not differ between the groups. Similarly, when comparing early- and late-arrest samples to the CG, a significant difference between groups was identified for PIA (ANOVA, *p* = 0.0285), but not for MGS. In Tukey’s post hoc test, PIA was significantly higher only in the early-arrest group compared with the CG (Figure 3b).

### 3.3. Tryptase Protein Expression

**Figure 5:**
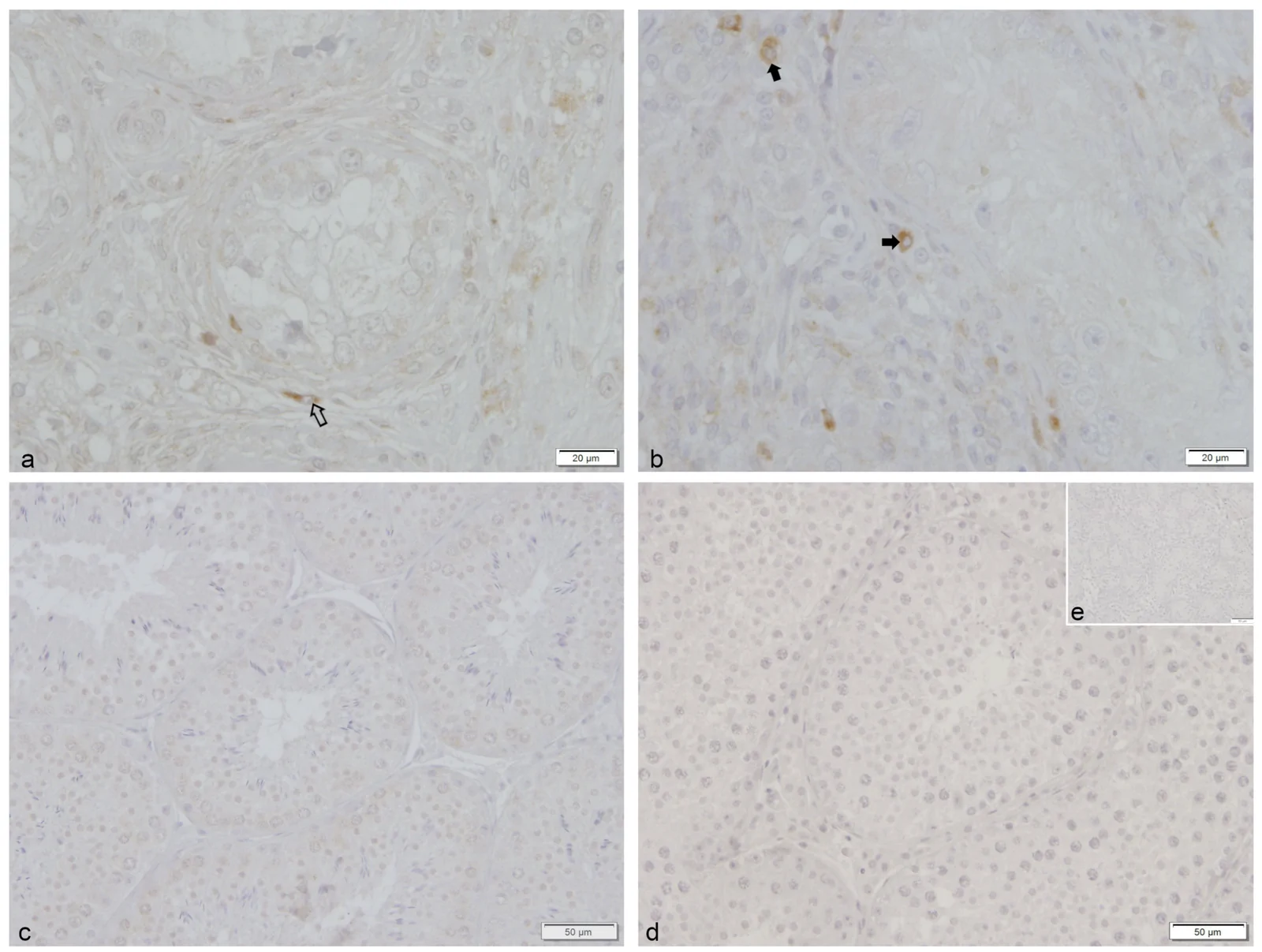
Immunostaining for tryptase in testicular samples from dogs with chronic asymptomatic orchitis (CAO) (**a**,**b**) and from normospermic dogs in the control group (CG) (**c**). CAO, (**c**) CG, (**d**) isotype control, (**e**) negative control (shown as an inset); Solid arrows (➡) indicate rounded mast cell, open arrows (⇨) indicate elongated mast cell ((**a**,**b**) 400× magnification, (**c**–**e**) 200× magnification).

Specific immunopositive staining for tryptase was only detected in the CAO samples, predominantly in the interstitial compartment, but was absent in the CG (Figure 5). Two types of immunopositive cells were identified: (1) mast cells with a typical rounded appearance, characteristically localized in the interstitial area, and (2) more elongated mast cells, typically located close to the tubular wall. Overall, the number of tryptase positive mast cells was low in the CAO group. Comparison of PTGS2- and tryptase-stained serial sections revealed that the majority of tryptase positive cells also stained immunopositive for PTGS2.

## 4. Discussion

We hypothesized that PTGS2 is involved in the pathophysiology of CAO in dogs. Since PTGS2 is the key enzyme in prostaglandin synthesis [14,15], we reasoned that PTGS2 might be upregulated in CAO compared to normal healthy testes obtained from normospermic canine patients. Our present data clearly support our hypothesis, as *PTGS2* mRNA expression was significantly higher in the CAO samples compared to the healthy canine testicular tissues. Moreover, the alteration in PTGS2 PIA was more pronounced in early-arrest CAO, indicating a correlation with the stage of spermatogenic arrest. Tryptase staining confirmed the presence of PTGS2-expressing mast cells solely in CAO group testes. The fact that StAR gene and protein expression levels were not decreased in CAO group testes indicates that the supply of cholesterol for steroid hormone synthesis is not reduced.

Even though alterations in the compartment distribution with a significantly increased interstitial and a decreased tubular compartment must be taken into consideration [10] when evaluating the mRNA expression levels, the increased *PTGS2* expression reported in rats with EAO is in good agreement with our results [17]. Similarly, the PTGS2-PIA was increased within the tubuli as well as in the interstitial area of CAO samples, supporting the hypothesis that PTGS2 is involved in testicular inflammatory changes. Regarding the cellular localization, a specific immunopositive signal against PTGS2 was identified in Sertoli, peritubular, and Leydig cells in both the CAO group and normal healthy testes. This is consistent with previous descriptions of healthy canine testicular tissue [25] and has also been described for healthy testes of Syrian hamsters [20], rats [22,30], and mice [21], as well as for infertile men [18,48]. Additionally, as confirmed by tryptase staining, most mast cells stained immunopositive for PTGS2. However, these cells were only identified in CAO-affected dogs, not in healthy controls, thus supporting their role in impaired spermatogenesis, as previously shown in infertile men [31,33,34]. Similar to the findings in the human cases [33,34], two morphologically different tissue types of tryptase-positive mast cells were identified, round and elongated mast cells, though their exact function and role remain unspecified up until now. In particular, peritubular mast cells were increased in infertile human patients compared to those with normal spermatogenesis [38,39], which matches the findings in our CAO-affected dogs. Studies on mast cells in the canine testis are sparse, and the results remain slightly conflicting [40,49]. Specifically, Anton et al. 1998 reported an absence of mast cells using toluidine blue and alcian blue staining [49], whereas our group previously identified sparse numbers using toluidine blue staining [40]. As mast cell “profiles”, namely protease expression levels, differ between anatomical sites [50] and potentially the pathological state of the site (healthy versus inflamed), comparative studies using mast-cell-specific antibodies and traditional stains might be suitable to further characterize canine testicular mast cells in CAO. As our study identified PTGS2-expressing mast cells in CAO, future studies should further investigate their role in CAO pathophysiology and determine whether specific PTGS2 inhibitors might reduce their presence or the progression of fibrosis.

Beyond cellular localization, we also elucidated the role of PTGS2 separately in the interstitial and tubular compartments by quantifying protein expression via objective computer-assisted analysis. Due to the cellular expression patterns, it can be concluded that expression in the interstitial compartment mainly represents PTGS2 expression in Leydig and peritubular cells, whereas expression in the tubular compartment represents Sertoli cell PTGS2 expression. Both PIA and MGS were significantly higher in the interstitial compartment in CAO samples compared to normal healthy testes, supporting the hypothesis of a higher PTGS2 expression due to an increased number of stained cells and stronger immunopositive staining in CAO-affected dogs. This observation is in good agreement with the *PTGS2* gene expression results. In EAO rats, *PTGS2* gene expression was correlated with the severity and progression of the disease [17]; likewise, PTGS2 protein expression (PIA and MGS) was positively associated with the degree of spermatogenic impairment. As PTGS2 is associated with testicular fibrosis [32], increased fibrosis and thickening of the basement membrane in CAO [7,10] might be related to the increased PTGS2 expression, thereby playing a role in disruption of spermatogenesis. Nevertheless, patients are only presented for confirmation of CAO after its clinical onset. Due to the clinically asymptomatic progression of the disease, ethical concerns, as well as the reluctance of owners, serial biopsies of affected dogs were not accessible. These would be essential to determine whether PTGS2 is involved in the development, maintenance, and/or progression of CAO.

Beyond the role of PTGS2 in inflammation, we aimed to further investigate the recently postulated effect of PTGS2 on steroidogenesis (or vice versa) in the canine testis [25]. In previous studies, we showed that PTGS2 [25] and StAR [29] expression levels were highest during the re-establishment of steroidogenesis following removal of the GnRH agonist implant. Using serial sections, we identified a significantly increased StAR and PTGS2 co-expression in CAO group samples, with immunopositive signals detected in Leydig and peritubular cells. This is, however, in contrast to the indirect inhibitory effect of increased PTGS2 on StAR and steroidogenesis as observed in murine Leydig cell cultures [28] during inflammation and during aging [30], which results in low testosterone concentrations. The increased PIA for StAR is especially interesting. As it seems unlikely that the number of Leydig cells changes postpubertally or in relation to CAO, it might instead indicate a change in Leydig cell size. Nevertheless, the fact that StAR was not downregulated in the CAO group supports the conclusion that spermatogenesis, but not steroidogenesis, is impaired in CAO in dogs. Furthermore, blood testosterone concentrations are not significantly altered in patients with CAO. Consequently, it can only be concluded that the first step of testicular steroidogenesis, the provision of cholesterol by StAR as part of the transduceosome, is not downregulated. To investigate the complete steroidogenic pathway, analysis via RNA-sequencing, proteomics, and metabolomics is needed [51].

Our study has several limitations: First, the results were obtained using small sample sizes and need to be verified in further studies. The investigated dogs belonged to various breeds, which influenced body weight and led to a high variability in body weight across cohorts. Additionally, the CG dogs were slightly younger than the CAO group dogs. This is, however, not necessarily a bias, as the exact onset of the disease remains unknown. Due to the asymptomatic nature of CAO, the disease was first recognized through unsuccessful matings. Age- and breed-matched controls should be included in further studies. Lastly, we did not quantify the total number of mast cells. Therefore, possible differences between early- and late-arrest of spermatogenesis could not be identified. The lack of serial biopsies during the course of the disease prevents the determination of whether PTGS2 is involved in the initiation, progression, or maintenance of CAO.

Nevertheless, our findings regarding increased PTGS2 expression associated with inflammation indicate that the administration of PTGS2 inhibitors could be a suitable therapeutic approach to suppress the testicular inflammation. In the healthy testis, contradictory results concerning the effects of non-steroidal anti-inflammatory drug treatment on spermatogenesis have been reported [52,53,54]. Specifically, oral acetylsalicylic acid or indomethacin as well as subcutaneous diclofenac treatment enhanced murine and canine spermatogenesis [52,53]; however, intraperitoneal acetylsalicylic acid decreased rat Leydig cell function and sperm counts [54]. Similar controversial effects of selective PTGS2 inhibitors have been described with short-term nimesulide treatment exerting no negative effects, whereas long-term treatment revealed nuclear fragmentation, vacuolated mitochondria and immature giant spermatids [21]. Daily celecoxib treatment over five weeks did not adversely affect testis weight or androgen levels, nor did it result in histological changes in the testes of rats [55]. The observed increase in interstitial testicular fluid, however, led to the assumption that damage could occur in case of pre-existing subfertility, though not in acute inflammatory diseases [55]. Nevertheless, our results indicate that short-term administration of selective PTGS2 inhibitors may be beneficial for reducing inflammation and potentially preventing CAO progression. A major limitation of this approach is that an early start of treatment requires the detection and diagnosis of the testicular insult at an early stage, possibly even before the manifestation of CAO, in order to be successful. Currently, such biomarkers are lacking.

## 5. Conclusions

Overall, our study confirms the significance of PTGS2 for CAO, as the PTGS2 gene and protein expression levels were significantly higher in patients with CAO, supporting its involvement in inflammation. Furthermore, mast cells coexpressing PTGS2 and tryptase were identified solely in CAO-affected testes. StAR expression was not reduced in CAO, ensuring an undisturbed supply of cholesterol for testicular steroidogenesis, although the complete subsequent synthetic cascade and the influence of PTGS2 thereon remain to be further elucidated. As these results implicate that selective PTGS2 inhibitors might offer a therapeutic option for CAO treatment, further studies on their potential benefits are urgently needed.

## Figures and Tables

**Table 1:** Sequences of primers for RT-qPCR, amplicon length in base pairs (bp), efficiency, and accession number (for = forward, rev = reverse).

Gene	Oligonucleotide Sequence (5′–3′)	Amplicon Length (bp)	Efficiency	Accession Number
*GAPDH*	for: GGCCAAGAGGGTCATCATCTC rev: GGGGCCGTCCACGGTCTTCT	228	1.96	NM_001003142.1
*ACTB*	for: GCTGTGCTGTCCCTGTATG rev: GCGTACCCCTCATAGATGG	98	1.92	NM_001195845.3
*PTGS2*	for: GGAGCATAACAGAGTGTGTGATGTG rev: AAGTATTAGCCTGCTCGTCTGGAAT	88	2.21	NM_001003354
*StAR*	for: CGAGGCTCCACCTGTGTGT rev: CCTTTCTGCTCAGGCATCTC	65	1.94	NM_001097542

**Table 2:** PTGS2 mRNA (ratios) and protein expression (percentage of immunopositive area, PIA, and mean gray scale, MGS) in testicular specimens collected from 11 dogs with chronic asymptomatic orchitis (CAO) and five normospermic dogs in the control group (CG). *PTGS2* mRNA ratios and all MGS results are presented as arithmetic mean ± standard deviation, whereas all PIA values are displayed as geometric mean (geometric standard deviation factor). CAO was compared with CG using unpaired *t*-tests; log10 transformation was applied to PIA values prior to analysis, and Welch’s correction was used for mRNA ratios.

Compartment	Analysis Level	CAO	CG	*p*-Value
	mRNA (ratios)	4.93 ± 1.72	1.15 ± 0.41	<0.0001
tubuli	PIA	1.05 (3.05)	0.07 (3.33)	0.0006
	MGS	98.16 ± 0.76	97.36 ± 0.32	0.0413
interstitium	PIA	1.89 (2.02)	0.06 (2.56)	<0.0001
	MGS	114.77 ± 0.86	113.07 ± 1.15	0.0051

**Table 3:** StAR mRNA (ratios) and protein expression (percentage of immunopositive area, PIA, and mean gray scale, MGS) in testicular specimens collected from dogs with chronic asymptomatic orchitis (CAO) and normospermic dogs in the control group (CG). MGS results are presented as arithmetic mean ± standard deviation, whereas StAR mRNA ratios and PIA values are displayed as geometric mean (geometric standard deviation factor). The CAO group was compared with the CG using unpaired *t*-tests; log10 transformation was applied to StAR mRNA ratios and PIA values prior to analysis, and Welch’s correction was used for mRNA ratios.

Compartment	Analysis Level	CAO	CG	*p*-Value
	mRNA (ratio)	2.92 (2.87)	0.78 (1.31)	0.0034
interstitium	PIA	1.79 (1.72)	0.35 (2.92)	0.0010
	MGS	99.36 ± 0.60	99.68 ± 0.75	0.3770

## Data Availability

The data presented in this study are available on request from the corresponding author. Further clinical data are not presented due to privacy reasons.

## Supplementary Materials

The following supporting information can be downloaded at: https://www.mdpi.com/article/10.3390/vetsci13101110/s1. Table S1: Baseline characteristics (breed, age in years, body weight in kg) of the individual patients suffering from chronic asymptomatic orchitis (CAO) and control group (CG) dogs with normospermic semen samples and unaffected spermatogenesis. The status of spermatogenesis (normal, early arrest, or late arrest) as well as immune cell infiltration (focal or generalized) are also presented. Additional details about the dogs with CAO are provided in [7].

## Abbreviations

The following abbreviations are used in this manuscript:

ACTB	β-Actin
ANOVA	Analysis of Variance
CAO	Chronic Asymptomatic Orchitis
CG	Control Group
EAO	Experimentally Induced Autoimmune Orchitis
GAPDH	Glyceraldehyde-3-Phosphate Dehydrogenase
MGS	Mean Gray Scale
bp	Base Pairs
PBS	Phosphate-Buffered Saline
PIA	Percentage of Immunopositive Area
PTGS2	Prostaglandin Endoperoxide Synthase 2
RT-qPCR	Reverse Transcription Quantitative Polymerase Chain Reaction
StAR	Steroidogenic Acute Regulatory Protein
