# Supplementary material for: Prostaglandin-Endoperoxide Synthase 2 (PTGS2) Expression Is Increased in Chronic Asymptomatic Orchitis in the Dog
Source: Vet Sci. 2026 Oct 17;13(10):1110. doi: 10.3390/vetsci13101110 (PMC13709730; doi:10.3390/vetsci13101110)
Supplement: Supplementary file 1 [file vetsci-13-01110-s001.zip › vetsci-4585627-supplementary.pdf]

Supplementary Table S1: Baseline characteristics (breed, age in years, body weight in kg) of the individual patients suffering from chronic asymptomatic orchitis (CAO), and control group (CG) dogs with normospermic semen samples and unaffected spermatogenesis. The status of spermatogenesis (normal, early arrest, or late arrest) as well as immune cell infiltration (focal or generalized) are also presented. Additional details about the CAO dogs are provided in [7].

No.	Group	Breed	Age	Body weight (kg)	Spermatogenesis (arrest/normal)	Presence of immune cells
1	CAO	Collie	5.7	24.8	early	generalized
2	CAO	Cairn Terrier	5.3	*	late	generalized
3	CAO	Collie	8.5	*	late	focal
4	CAO	Miniature Poodle	7.7	5.0	late	generalized
5	CAO	Labrador Retriever	4.5	35.0	late	focal
6	CAO	Icelandic sheepdog	9.5	14.3	early	generalized
7	CAO	Welsh Corgie Pembroke	4.7	15.0	early	generalized
8	CAO	Collie	5.8	*	early	generalized
9	CAO	Coton de Tulear	6.0	6.0	late	focal
10	CAO	Beagle	5.8	18.0	late	focal
11	CAO	Parson Russell Terrier	5.3	7.7	early	generalized
A	CG	Havanese	1.3	5.6	normal	n.a.
B	CG	Boston Terrier	1.6	6.3	normal	n.a.
C	CG	Mongrel	8.8	11.5	normal	n.a.
D	CG	Chihuahua	0.9	3.6	normal	n.a.
E	CG	Maltese	9.9	11	normal	n.a.

*: not recorded
n.a.: not applicable
